# A novel positive end-expiratory pressure titration using electrical impedance tomography in spontaneously breathing acute respiratory distress syndrome patients on mechanical ventilation: an observational study from the MaastrICCht cohort

**DOI:** 10.1007/s10877-024-01212-8

**Published:** 2024-08-28

**Authors:** S.J.H. Heines, S.A.M. de Jongh, F.H.C. de Jongh, R.P.J. Segers, K.M.H. Gilissen, I.C.C. van der Horst, B.C.T. van Bussel, D.C.J.J. Bergmans

**Affiliations:** 1 Department of Intensive Care, Maastricht University Medical Center+, P. Debyelaan 25, P.O. Box 5800, Maastricht, 6202 AZ The Netherlands; 2 Department of Pulmonology, Medisch Spectrum Twente, Enschede, The Netherlands; 3 Cardiovascular Research Institute Maastricht (CARIM), Maastricht University, P.O. Box 616, Maastricht, 6200 MD The Netherlands; 4 Care and Public Health Research Institute (CAPHRI), Maastricht University, P.O. Box 616, Maastricht, 6200 MD The Netherlands; 5 School of Nutrition and Translational Research in Metabolism (NUTRIM), Maastricht University, P.O. Box 616, Maastricht, 6200 MD The Netherlands

**Keywords:** Electrical impedance tomography, ARDS, Spontaneous ventilation, Positive end-expiratory pressure, Pendelluft, Airway occlusion pressure, Regional peak flow

## Abstract

There is no universally accepted method for positive end expiratory pressure (PEEP) titration approach for patients on spontaneous mechanical ventilation (SMV). Electrical impedance tomography (EIT) guided PEEP-titration has shown promising results in controlled mechanical ventilation (CMV), current implemented algorithm for PEEP titration (based on regional compliance measurements) is not applicable in SMV. Regional peak flow (RPF, defined as the highest inspiratory flow rate based on EIT at a certain PEEP level) is a new method for quantifying regional lung mechanics designed for SMV. The objective is to study whether RPF by EIT is a feasible method for PEEP titration during SMV. Single EIT measurements were performed in COVID-19 ARDS patients on SMV. Clinical (i.e., tidal volume, airway occlusion pressure, end-tidal CO_2_) and mechanical (cyclic alveolar recruitment, recruitment, cumulative overdistension (OD), cumulative collapse (CL), pendelluft, and PEEP) outcomes were determined by EIT at several pre-defined PEEP thresholds (1–10% CL and the intersection of the OD and CL curves) and outcomes at all thresholds were compared to the outcomes at baseline PEEP. In total, 25 patients were included. No significant and clinically relevant differences were found between thresholds for tidal volume, end-tidal CO_2_, and P0.1 compared to baseline PEEP; cyclic alveolar recruitment rates changed by -3.9% to -37.9% across thresholds; recruitment rates ranged from − 49.4% to + 79.2%; cumulative overdistension changed from − 75.9% to + 373.4% across thresholds; cumulative collapse changed from 0% to -94.3%; PEEP levels from 10 up to 14 cmH_2_O were observed across thresholds compared to baseline PEEP of 10 cmH_2_O. A threshold of approximately 5% cumulative collapse yields the optimum compromise between all clinical and mechanical outcomes. EIT-guided PEEP titration by the RPF approach is feasible and is linked to improved overall lung mechanics) during SMV using a threshold of approximately 5% CL. However, the long-term clinical safety and effect of this approach remain to be determined.

**Supplementary Information:**

The online version contains supplementary material available at 10.1007/s10877-024-01212-8.

## Background

Previous studies have suggested that vigorous spontaneous breathing efforts during spontaneous mechanical ventilation (SMV) in acute respiratory distress syndrome (ARDS) may inflict or worsen the lung injury that is already present through pendelluft (i.e., air shift between lung regions due to a pressure gradient caused by temporal and spatial differences) and cyclic alveolar recruitment (i.e., alveoli opening at end-inspiration and closing during end-expiration, also known as atelectrauma) [1–10]. This phenomenon is called patient self-inflicted lung injury [11].

One promising preventive therapy for vigorous spontaneous breathing efforts dependent on lung injury in SMV modes includes applying higher positive end expiratory pressure (PEEP) levels compared to current clinical practice. This may decrease the magnitude of the spontaneous effort and may improve lung ventilation homogeneity by opening up partially closed/open lung tissue (open lung concept), suggesting that this may lead to less injurious ventilation [12, 13]. In contrast, healthy lung tissue may be overdistended when PEEP levels are set too high, inducing ventilator-induced lung injury [14]. As a result, optimal titration of PEEP is necessary.

Currently, there is no universally accepted approach for personalized PEEP strategies in SMV [15]. Most strategies in these ventilatory modalities are partially subjective and based on global lung parameters, such as the airway occlusion pressure (which is considered a surrogate for work of breathing), tidal volumes, and oxygenation status (i.e., arterial blood gas and peripheral oxygen saturation) [16–18]. This approach may ultimately lead to suboptimal ventilatory strategies and potentially unnecessary lung injury [19]. PEEP titration by EIT in ARDS on controlled mechanical ventilation has shown promising results regarding ventilator weaning success [20, 21]. Nonetheless, the EIT algorithm used in these studies does not apply to SMV. Therefore, our group recently developed and validated a similar EIT algorithm in ARDS patients on controlled mechanical ventilation based on regional peak flow (RPF, defined as the highest inspiratory flow rate based on EIT at a certain PEEP level) for quantifying regional overdistension and regional collapse [22]. The algorithm is developed in such a way that it is applicable in SMV. We believe that EIT has the potential to provide additional information regarding regional lung mechanical properties of heterogeneously affected ARDS lungs during SMV and simultaneously can be used to titrate PEEP in this population. Therefore, the objective is to study whether PEEP-titration by the RPF algorithm is feasible in SMV.

## Methods

### Population and EIT measurements

In the present study, data from the prospective Maastricht Intensive Care COVID-19 Cohort (MaastrICCht) were used, a comprehensive clinical variable collection of serial data on mechanically ventilated COVID-19 patients starting from March 2020 [23]. This study was approved by the local medical ethics review committee (METC: 2020 − 1565), registered in NTR with the number NL8613, and conducted in accordance with the Declaration of Helsinki (as revised in 2013). During the pandemic, the board of directors of the Maastricht UMC + adopted a policy to inform patients or their legal representative and ask for their consent to use their data for COVID-19 research purposes.

**Fig. 1:**
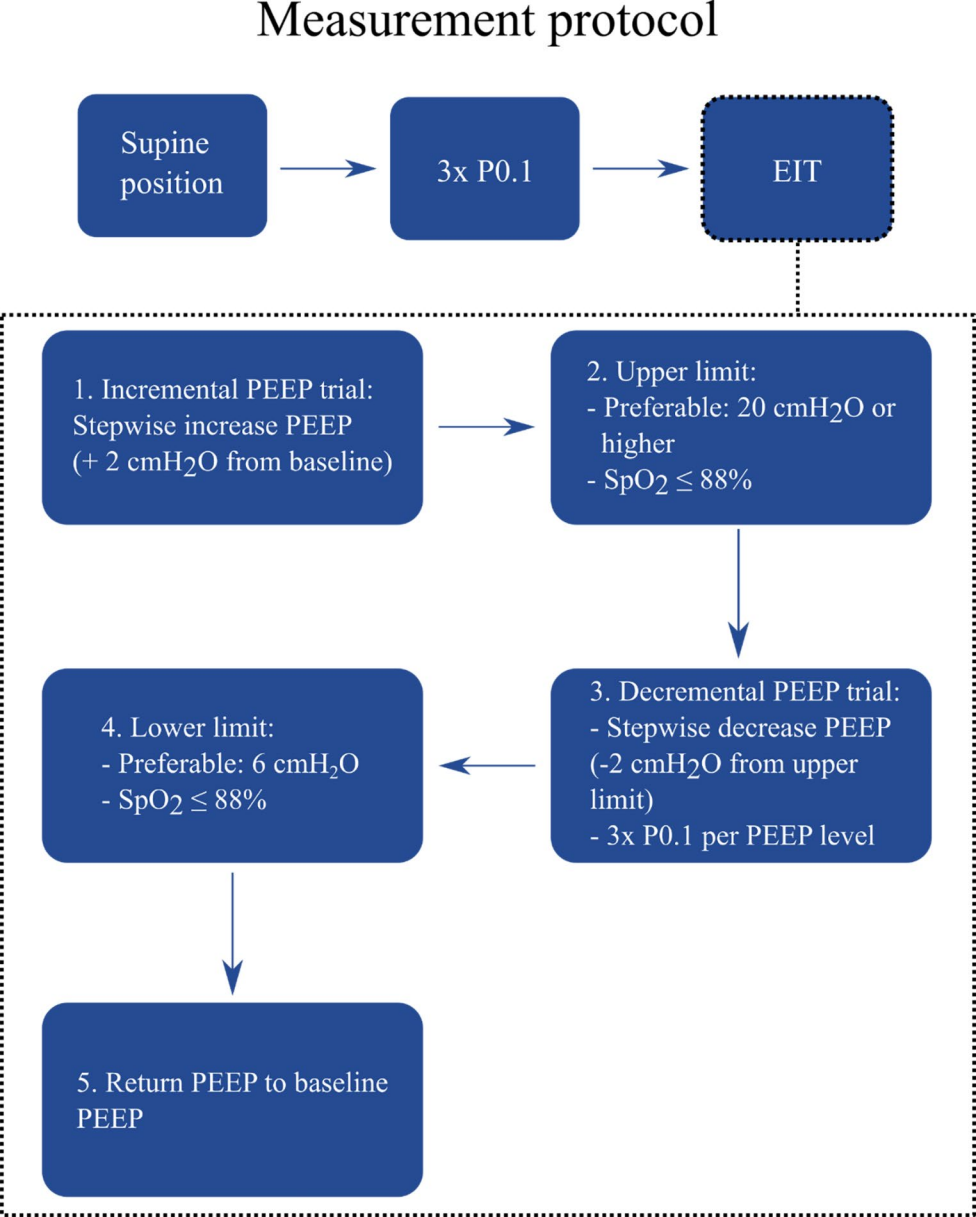
Measurement protocol for the SMV population. During the EIT measurement, only the PEEP-level is adjusted, while other respiratory settings (i.e., pressure support, slope and oxygen fraction) remain unchanged. EIT: electrical impedance tomography; PEEP: positive end-expiratory pressure; P0.1: airway occlusion pressure; SpO_2_: peripheral oxygen saturation

Within the serial data collection of the MaastrICCht cohort, adult COVID-19 patients with PCR-proven COVID-19 or a CO-RADS score of 4 or 5 who were mechanically ventilated by SMV (either by continuous positive airway pressure ventilation or pressure support ventilation) and met the Berlin criteria for ARDS between December 2020 and May 2021 were included in this study [24, 25]. For the present observational study, an EIT measurement was performed once during SMV. The EIT measurement protocol is visualized in Fig. 1 and comprises three pre-EIT airway occlusion pressure measurements (P0.1), which is a measure of work of breathing and an EIT measurement (as extensively described in the supplemental data of Tas et al.) [23, 26]. The EIT measurement was performed in a supine position and included an incremental PEEP trial and a subsequent decremental PEEP trial in steps of 2 cmH_2_O. Each PEEP step took approximately 30 s. After finishing the EIT measurement, the PEEP level was returned to the baseline (i.e., the PEEP level determined by the attending physician before the EIT measurement, based on clinical information like respiratory system compliance, PaO_2_/FiO_2_-ratio, BMI, hemodynamics, etc.).

### Data acquisition and pre-processing

The EIT measurements were recorded with the Pulmovista 500 (Dräger Medical, Lübeck, Germany) with a sample frequency of 50 Hz. Raw EIT data files from the Pulmovista 500 (i.e., raw relative impedances with a spatial resolution: 32 × 32) were converted to MATLAB (the MathWorks Inc., Natick, MA, USA) readable files using Dräger’s “Data Analysis Tool 6.3”. Raw ventilator data were converted to MATLAB readable files using Dräger’s “EITeasy” analysis tool. Cardiac artifacts in the raw EIT data were then filtered using a bi-directional fourth order 0.83 Hz low-pass Butterworth filter using MATLAB R2021a.

### Outcome measures

Mechanical outcomes were calculated based on raw EIT data and included cumulative overdistension (OD) and collapse (CL) rates based on a regional peak flow approach, as studied previously by our group [22]. Other mechanical outcomes such as pendelluft [10, 27], recruitment rates, and Cyclic alveolar recruitment (CAR) rates [28], were also calculated based on raw EIT data as described by Liu et al. and Sang et al. []. Clinical outcomes were derived from the ventilator, such as tidal volume per kilogram predicted body weight (TV), end-tidal carbon dioxide (etCO_2_), and the P0.1. For each decremental PEEP step, the median outcome value was calculated for all outcome measures. All outcome measures calculations were performed in MATLAB R2021a.

### Pre-defined thresholds

**Fig. 2:**
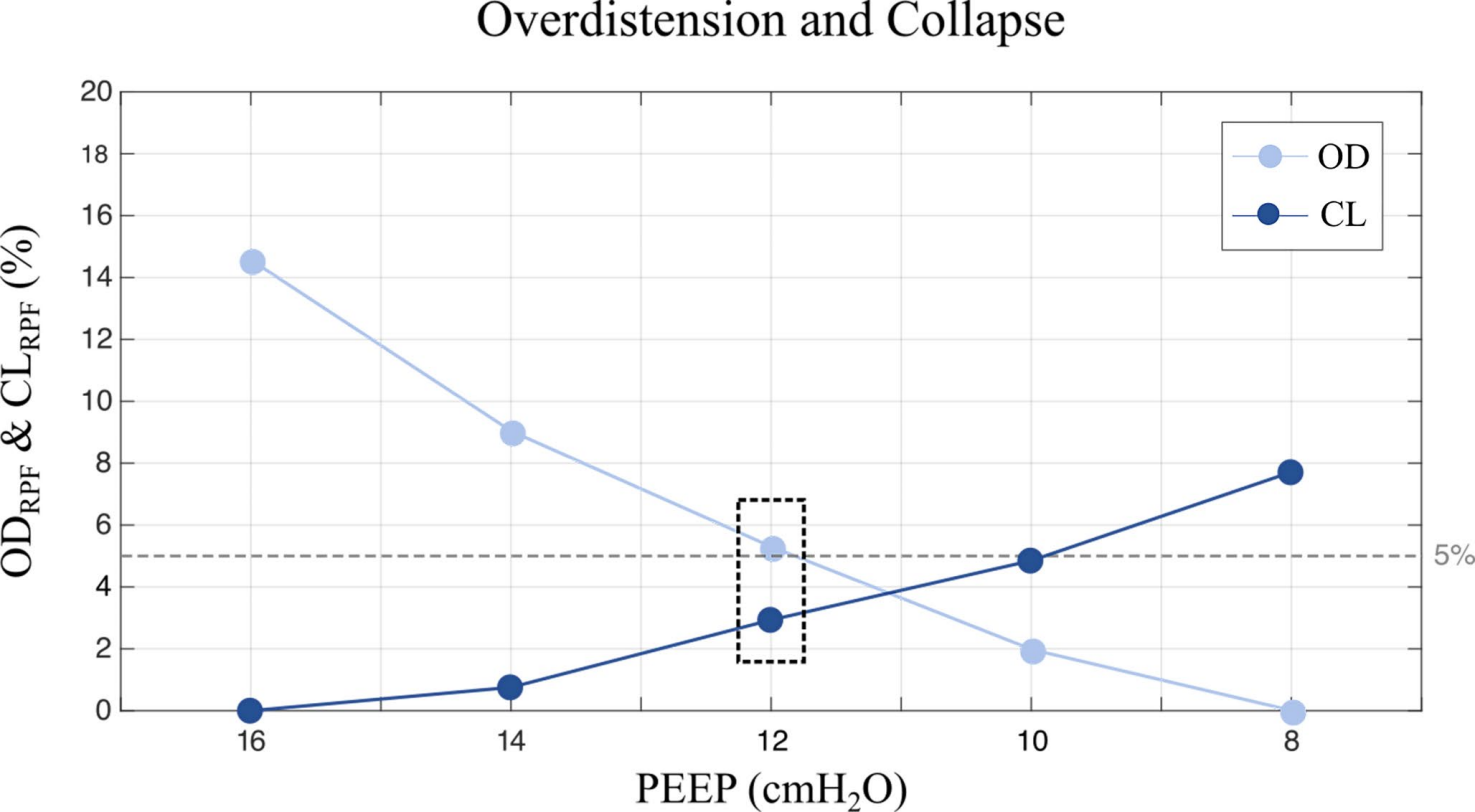
Example of determining optimal positive end-expiratory pressure based on two pre-defined thresholds: 5% cumulative collapse and the intersection of the cumulative overdistension rate and cumulative collapse rate curves. The grey dashed line represents the 5% cumulative collapse rate threshold. For this approach a positive end expiratory pressure level of 10 is chosen, since its cumulative collapse rate value is closest and under the 5% cumulative collapse rate of all positive end expiratory pressure levels during the decremental trial. An identical approach is used for the 1–4% and 6–10% cumulative collapse threshold. The intersection of the cumulative collapse and cumulative overdistension is considered the positive end expiratory pressure level, where the difference between the cumulative overdistension rate and cumulative collapse rate is the lowest. In this example, the intersection is found at a positive end expiratory pressure level of 12 (black dashed rectangle). CL: cumulative collapse rate; OD: cumulative overdistension rate; PEEP: positive end expiratory pressure

A threshold indicates a maximum tolerated value used to make decisions about for example a certain therapy, treatment or setting, The outcome measures, as mentioned above, were calculated at multiple pre-defined thresholds and compared to the outcome measures at the PEEP level during the decremental trial equal to the baseline PEEP (i.e., the PEEP level determined by the attending physician before the EIT measurement). Some groups use the intersection of the OD and CL curve, which provides a compromise between collapsed and overdistended lung tissue, or use the highest dynamic compliance to titrate PEEP [29]. We use a maximum of 5% cumulative collapse approach in controlled mechanical ventilation. Due to this lack of consensus, the examined pre-defined “optimal” PEEP thresholds in the present study are defined as the intersection of the OD and CL curve and a limit of 1% CL to 10% CL. For example, Fig. 2 visualizes the 5% CL and intersection thresholds for an arbitrary patient. The blue dashed line represents the 5% CL limit, indicating that this amount of CL is accepted. As a result, the optimal PEEP for this threshold is 10 cmH_2_O. In the present study, the intersection is considered the PEEP level, where the difference between the OD and CL rates is the lowest. Therefore, the optimal PEEP level for this approach in this example is equal to 12 cmH_2_O (magenta rectangle). In the present study, feasibility of EIT-based PEEP titration by RPF in SMV is acknowledged when an EIT-based threshold by RPF is found, which yields an optimum compromise between several lung mechanics parameters, as assessed by EIT, and clinical parameters.

### Statistical analysis

Patient characteristics at intensive care unit (ICU) admission and ventilation characteristics are presented as median with interquartile ranges for continuous variables, whereas categorical variables are presented in frequencies (%). In the present study, the day of the EIT measurement after intubation is calculated from the moment of the first intubation, while the EIT measurement day in spontaneous mechanical ventilation is calculated from the moment the patient is switched to SMV or when the patient is admitted to the ICU in an SMV mode. To address potential selection bias, admission variables of the present study’s subcohort are compared to those from all MaastrICCht cohort patients who were mechanically ventilated but did not receive EIT during SMV between March 2020 and March 2021 by the Mann-Whitney-U test and Chi-square test. Outcome measures per pre-defined threshold were tested for normality using the Shapiro-Wilk test. Then, the outcomes over time for all pre-defined thresholds are compared to the baseline by either a repeated measures ANOVA or a Friedman test according to normality. When the model revealed a significant effect, Dunn’s post-hoc tests, including a Bonferroni correction, was applied to determine which outcomes at the multiple thresholds differed from baseline outcomes. In all tests, α is set at 0.05. The statistical analyses were performed in SPSS version 28 (IBM, Armonk, NY).

## Results

In total, 25 patients (80% male) were included in the present study and had a median age of 66 years and a BMI of 28.7 kg/m2 (Table 1). Median APACHE II and SOFA scores at ICU admission were 14.0 and 10.0 points and four patients suffered from known chronic lung disease prior to ICU unit admission. On average, the EIT measurements were performed ten days [6.5–25.5] after intubation and three days [2.0–4.0] after the start of spontaneous mechanical ventilation. There were no differences, except for pH (p-value = 0.049), between the subcohort of 25 patients in the present study, compared to the mechanically ventilated MaastrICCht cohort patients who were not subjected to SMV (a total of 269 inclusions, 232 of whom received any form of invasive mechanical ventilation and 207 of whom were not subjected to EIT during SMV) at admission (Table S1).

**Table 1:** Patient characteristics

**Patient characteristics** ^¶^ **(Median [Q1-Q3])**	
N	25
Age, year	66.0 [59.8–73.5]
Gender, men	20 (80.0%)
Body mass index, kg/m^2^	28.7 [26.4–30.4]
Chronic lung disease, N	4 (16.0%)
APACHE II score, points	14.0 [12.5–17.3]
SOFA score, points	10 [9.5–12.3]
Intubation, N	22 (88.0%)
FiO_2_, %	80 [50–100]
PaO_2_/FiO_2_, mmHg	13.0 [10.0–20.0]
PaO_2_, mmHg	9.5 [8.8–10.0]
PaCO_2_, mmHg	4.9 [4.5–5.8]
pH	7.48 [7.35–7.51]
Respiratory Rate, min^− 1^	26 [23–29]
Mean Arterial Pressure, mmHg	107 [87–122]
Day of EIT measurement after intubation^†^, days	10.0 [6.5–25.5]
Day of EIT measurement in spontaneous mechanical ventilation^‡^, days	3.0 [2.0–4.0]
**Baseline ventilation variables** ^§^ **(Median [Q1-Q3])**	
Oxygen fraction (FiO_2_), %	40 [30–55]
Positive End-Expiratory Pressure (PEEP), cmH_2_O	10 [8–12]
Pressure Support, cmH_2_O	2 [0–6]
PaO_2_/FiO_2_-ratio, mmHg	195 [146–263]
Arterial blood gas oxygen (paO_2_), mmHg	76.5 [71.3–86.8]
Arterial blood gas carbon dioxide (paCO_2_), mmHg	42.0 [37.3–46.5]
pH	7.46 [7.44–7.49]

^¶^At ICU admission, ^**†**^ Day 1 is considered the first day of the first intubation period ^‡^ Day 1 is considered the first day after the last period of controlled mechanical ventilation or the first day of intubation when the patient is admitted to the intensive care unit by a spontaneous mechanical ventilation mode; ^§^ Ventilator settings and arterial blood gas variables prior to the EIT measurement. APACHE: Acute Physiology And Chronic Health Evaluation; ICU: Intensive Care Unit; SOFA: Sequential Organ Failure Assessment

**Fig. 3:**
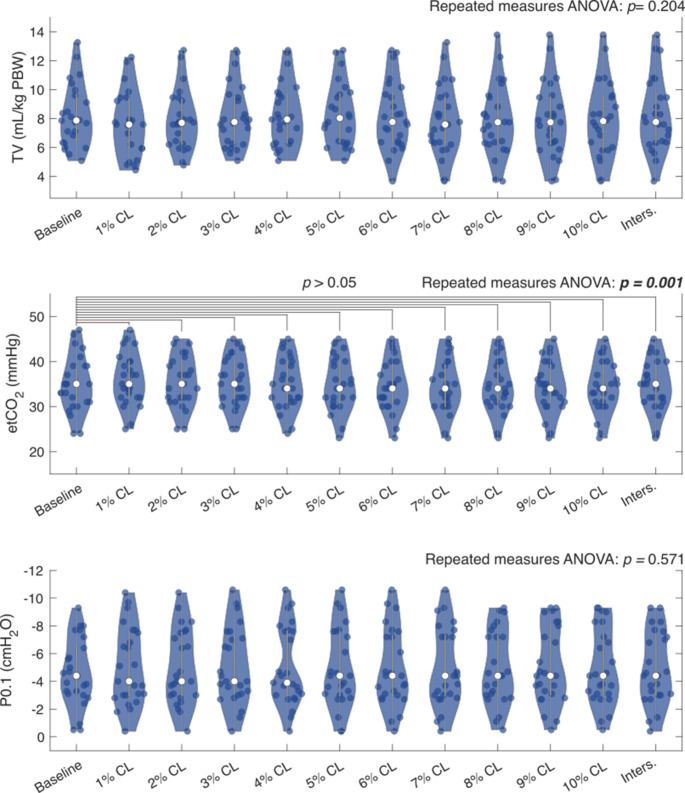
Median clinical outcomes at the PEEP determined by the pre-defined thresholds and at baseline PEEP. The repeated measures ANOVA results are showed per variable. If a significant outcome is found in the repeated measures ANOVA, post hoc analysis with Bonferroni correction is visualized for each pre-defined threshold and baseline. Significant differences in the post hoc analysis are colored red. ANOVA: analyses of variances; CL: cumulative collapse rate; etCO_2_: end-tidal carbon dioxide; Inters.: intersection of cumulative overdistension and collapse curves; P0.1: airway occlusion pressure; PEEP: positive end-expiratory pressure; TV: tidal volume per kilogram of predicted body weight

No significant differences were found between baseline tidal volume (7.9 mL/kg predicted body weight [6.3 mL/kg – 9.7 mL/kg]) and all threshold outcomes (Repeated measures ANOVA: *p* = 0.204). For etCO_2_, repeated measures ANOVA (*p* = 0.001) and subsequent post hoc analysis without Bonferroni correction revealed a significant difference between the 1% CL threshold [32 cmH_2_O – 40 cmH_2_O] and baseline (35 mmHg [31 mmHg – 39 mmHg]). However, with Bonferroni correction, this significant difference dissolved (*p* > 0.05). The other pre-defined thresholds also remained statistically insignificant (*p* > 0.05). For P0.1, no significant differences were found between baseline (-4.4 cmH_2_O [-6.6 cmH_2_O – -3.1 cmH_2_O]) and the pre-defined thresholds (repeated measures ANOVA: *p* = 0.571) (Fig. 3).

**Fig. 4:**
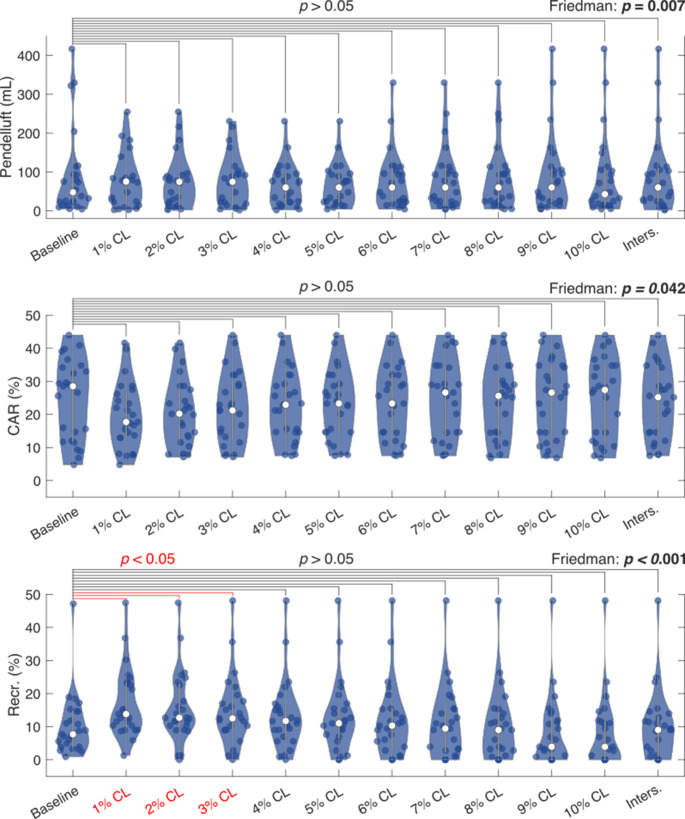
Median mechanical outcomes at the PEEP determined by the pre-defined thresholds and at baseline PEEP. The Friedman test results are showed per variable. If a significant outcome is found in the Friedman test, post hoc analysis with Bonferroni correction is visualized for each pre-defined threshold and baseline. Significant differences in the post hoc analysis are colored red. ANOVA: analyses of variances; CAR: Cyclic alveolar recruitment; CL: cumulative collapse rate; Inters.: intersection of cumulative overdistension and collapse curves; Recr.: Recruitment rate

Concerning pendelluft, the Friedman test was significant (*p* = 0.007), but post hoc analysis revealed no significant differences between baseline (48 mL [19 mL – 103 mL]) and the pre-defined thresholds (*p* > 0.05). Identical results were found for CAR, where baseline was equal to 28.5% [11.9 − 34.5%] (Friedman test: *p* = 0.042; post hoc: *p* > 0.05). Recruitment rates at 1% CL to 3% CL (1% CL: 13.8% [9.7 − 23.3%], *p* < 0.001; 2% CL: 12.7% [9.7 − 23.1%], *p* < 0.001; 3% CL: 12.5% [9.0 − 18.7%], *p* = 0.005) were significantly higher compared to baseline (7.7% [3.9 − 14.7%]) (Friedman test: *p* < 0.001) (Fig. 4).

**Fig. 5:**
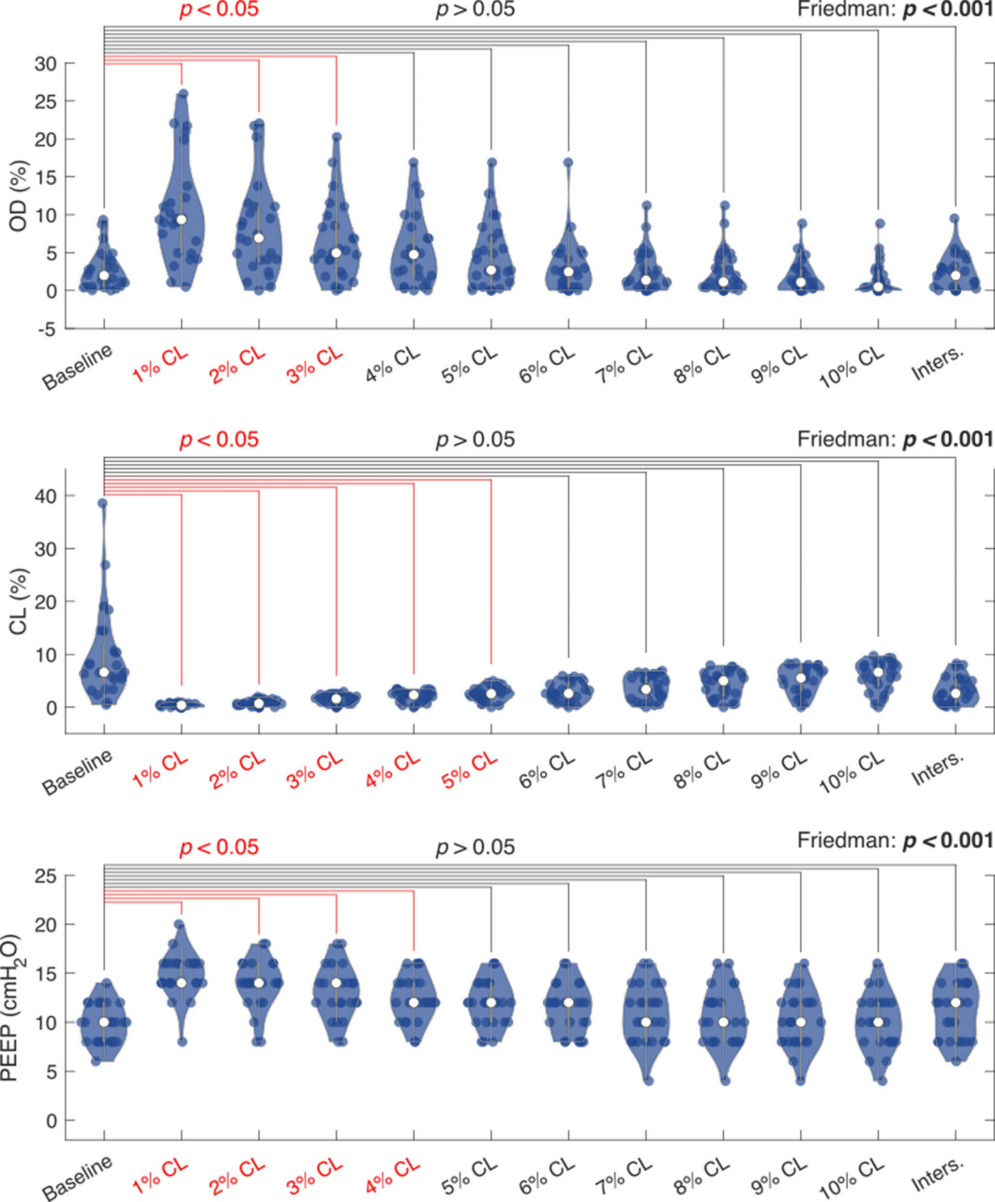
Median mechanical outcomes at the PEEP determined by the pre-defined thresholds and at baseline PEEP. The Friedman test results are showed per variable. If a significant outcome is found in the Friedman test, post hoc analysis with Bonferroni correction is visualized for each pre-defined threshold and baseline. Significant differences in the post hoc analysis are colored red. CL: cumulative collapse rate; etCO_2_: end-tidal carbon dioxide; Inters.: intersection of cumulative overdistension and collapse curves; OD: cumulative overdistension rate; P0.1: airway occlusion pressure; PEEP: positive end-expiratory pressure; TV: tidal volume per kilogram of predicted body weight

The 1% CL to 3% CL thresholds also showed significantly increased OD (1% CL: 9.3% [4.9 − 13.0%], *p* < 0.001; 2% CL: 6.9% [3.6 − 11.1%], *p* < 0.001; 3% CL: 5.0% [2.2 − 9.2%], *p* = 0.005) compared to a baseline of (1.9% [0.5 − 4.0%]) (Friedman test: *p* < 0.001). For CL, the 1% CL to 5% CL threshold were significantly lower (1% CL: 0.4% [0.0 − 0.7%], *p* < 0.001; 2% CL: 0.7% [0.2 − 1.4%], *p* < 0.001; 3% CL: 1.6% [0.7 − 2.4%], *p* < 0.001; 4% CL: 2.3% [1.1 − 3.1%], *p* = 0.001; 5% CL: 2.6% [1.5 − 4.8%], *p* = 0.009) compared to 6.6% [4.3 − 12.6%] at baseline (Friedman test: *p* < 0.001). Last, significantly increased PEEP levels were found at the 1% CL to 4% CL threshold (1% CL: 14 cmH_2_O [14 cmH_2_O – 16 cmH_2_O], *p* < 0.001; 2% CL: 14 cmH_2_O [13 cmH_2_O – 16 cmH_2_O], *p* < 0.001; 3% CL: 14 cmH_2_O [12 cmH_2_O – 14 cmH_2_O], *p* = 0.001; 4% CL: 12 cmH_2_O [11 cmH_2_O – 14 cmH_2_O], *p* = 0.027) compared to 10 cmH_2_O [8 cmH_2_O – 12 cmH_2_O] at baseline (Friedman test: *p* < 0.001) (Fig. 5).

## Discussion

The assumption of EIT compliance measurement is that pressure changes are uniform throughout the lung when flow values reaches zero at the end of inspiration, and expiration. This is not the case during SMV. Therefore, we designed another concept, namely the RPF for PEEP titration.

In our previous paper we described the concept of mechanical overdistension and collapse by EIT using a RPF method and validated this concept by comparing it to mechanical overdistension and collapse using the compliance method at multiple PEEP levels in a group of patients under controlled mechanical ventilation [22]. In the current manuscript we demonstrated that EIT-guided PEEP titration using the RPF approach is feasible in spontaneously breathing patients by performing a PEEP trial.

The main finding of the present study is that PEEP titration by EIT using the RPF algorithm seems feasible in a COVID-19-related ARDS cohort on SMV. It suggests that approximately 5% cumulative collapse is a surrogate for the optimal PEEP since it yields an optimal compromise of all mechanical and clinical outcomes taken into account in the present study. Notably, increased PEEP levels were found at approximately 5% cumulative collapse compared to baseline PEEP, as set by the clinician prior to EIT (12 cmH_2_O versus 10 cmH_2_O).

Effect of PEEP on clinical and mechanical outcomes.

As stated in a recent review by Yoshida et al., available evidence regarding vigorous spontaneous breathing efforts during mechanical ventilation and patient self-inflicted lung injury mostly appeared in laboratory studies but also in case series and a few clinical studies [13]. These studies are elaborated on in the following paragraphs. Combined, they suggest that higher PEEP appears associated with less injurious breathing efforts.

More specifically, in human studies, Morais et al. found that higher PEEP resulted in less inspiratory effort, expressed by decreased oesophageal pressure swings and peak pleural pressure swings [8]. Also, indirect proof of PEEP reducing inspiratory effort has been described by Patel et al. [30]. They compared two non-invasive ventilation approaches in ARDS patients (helmet versus face mask). The helmet could deliver higher PEEP levels which resulted in less spontaneous effort suggested by a lower respiratory rate. In the present study, the respiratory effort was expressed by P0.1 and was independent of the pre-defined thresholds, which does not align with the findings of Morais et al. However, no data on transpulmonary pressures and respiratory rate monitoring during EIT were collected in the present study. Therefore, no conclusive comparison between the studies can be made regarding the inspiratory effort. Also, Morais et al. measured respiratory effort five to ten minutes after the PEEP change, while in the present study, each PEEP level in the decremental PEEP trial only lasted 30 to 60 s. Unfortunately, in Patel et al., the timing of the respiratory rate measurements is unclear.

Coppadoro et al. studied the effects of different pressure support settings on several clinical and lung mechanics parameters between a “high pendelluft” and a ”low pendelluft” group on SMV [31]. They found that the amount of pendelluft decreases with increased pressure support during pressure support ventilation, while PEEP remained constant. However, the division of the EIT grid into four dorsal-ventral regions of interest is a major limitation. Calculating pendelluft this way may lead to underestimation because air may redistribute within one region of interest and may thus be missed. Nonetheless, tidal recruitment may not decrease due to higher ΔPsupp, since it may only open more atelectatic alveoli, which may collapse again at end-expiration. This may potentially result in greater lung injury. It is therefore believed that higher PEEP is a more potent approach to reducing vigorous breathing efforts during SMV. Interestingly, the present study shows an increase in average pendelluft when the CL threshold decreases. Simultaneously, in some patients, higher pendelluft in higher CL thresholds are found. This means that pendelluft may increase at lower and higher PEEP levels. Lower PEEP level pendelluft may represent an air shift from the non-overdistended non-dependent part of the lung towards the dependent lung fields, which are prone to CAR. The present study showed higher CAR rates when PEEP decreased. In contrast to lower PEEP, at higher PEEP levels, pendelluft may arise due to an air shift from overdistended non-dependent lung fields towards diseased dependent lung fields recruited by the higher PEEP. In the present study, both OD and recruitment rates increased with higher PEEP. However, pendelluft is a type of asynchronous alveolar ventilation, which is usually caused by different regional time constants or dynamic pleural pressure variations, people in healthy conditions might have pendelluft because the lung is inherently inhomogeneous, therefore pendelluft might be not harmful per se.

Regarding laboratory studies, Yoshida et al. showed that lower PEEP levels result in more pendelluft in a porcine model using EIT than higher PEEP levels [6]. This study determined the high PEEP level by EIT using Costa’s approach with a cumulative collapse rate < 3%, whereas the low PEEP level was defined as the high PEEP level minus 10 cmH_2_O. By computed tomography, they found that the CAR rate was higher at the low PEEP levels compared to the high PEEP levels. These results align with the present observations since pendelluft and CAR were elevated at higher CL thresholds and thus lower PEEP levels. Moreover, Yoshida et al. found a significant lower PaO_2_/FiO_2_-ratio at lower PEEP levels with spontaneous breathing efforts one hour after lung injury induction. Interestingly, at the same time, oesophageal pressure swings were less negative with high PEEP compared to low PEEP with spontaneous breathing efforts one hour after lung injury induction. This can be interpreted as a decrease in inspiratory effort. We could neither confirm nor refute the PaO_2_/FiO_2_ and oesophageal pressure results since we did not perform arterial blood gas analyses and oesophageal pressure measurements during the PEEP trial in the present study. Meanwhile, tidal volumes and arterial CO_2_ levels did not differ between low and optimal PEEP levels in the study mentioned above. This is in line with our findings since, on average, both tidal volume and end-tidal CO_2_ seem independent of the pre-defined thresholds assessed in this study. However, we only performed PEEP steps of approximately 30 s and cannot rule out any long-term CO_2_ or tidal volume changes due to too low PEEP levels.

Similar results were reported by Morais et al. in a rabbit and porcine ARDS model [8]. Histologic research in rabbits revealed that lower PEEP levels with spontaneous breathing efforts led to increased lung injury compared to higher PEEP with spontaneous breathing efforts. Also, delta transpulmonary pressures were significantly lower in higher PEEP with spontaneous breathing efforts after one hour, indicating decreased inspiratory effort during a breath. Tidal volumes and arterial CO_2_ levels were equal between high and low PEEP with spontaneous breathing efforts. In pigs, severe ARDS was induced by lung lavage followed by injurious mechanical ventilation. After that, high PEEP was determined by EIT, using a threshold at 1% cumulative collapse, whereas low PEEP was set according to the ARDSnet PEEP/FiO_2_-Table [32]. On average, the PaO_2_/FiO_2_-ratio in pigs was non-statistically higher in high PEEP compared to low PEEP with spontaneous breathing efforts and delta transpulmonary pressures were lower in high PEEP with spontaneous breathing. Like rabbits, pigs showed no differences in tidal volumes and arterial CO_2_ levels.

Magalhães et al. studied the effect of different PEEP levels on clinical and lung mechanical outcomes in a rat model but did not find a statistically significant difference in PaO_2_/FiO_2_-ratio, P0.1 and arterial CO_2_ between lower and higher PEEP in SMV [33]. This can be explained by the fact that only mild injury (i.e., administration of lipopolysaccharide intratracheally, suspended in a saline solution) was induced.

Quantifying CAR and recruitment using EIT has only recently been published by Liu et al. [28]. They studied the effect of multiple PEEP levels on CAR and recruitment rates in controlled mechanical ventilation in pigs. During an incremental PEEP trial, they reported decreased CAR rates and increased recruitment rates at higher PEEP levels. These results are in line with the finding in the present study, despite performing the analyses on the PEEP levels in a decremental PEEP trial in humans on SMV.

## Limitations

At ICU admission, the inclusions (i.e., patients who received EIT in SMV) of the present study did not differ from the rest of the invasive mechanically ventilated cohort (i.e., patients who did not receive EIT during SMV or did not receive EIT at all) in terms of age, gender, BMI, chronic lung disease history, APACHE II score and SOFA score. This suggests that our results in 25 patients are generalizable to the full Maastricht Intensive Care COVID-19 Cohort and possibly mechanically ventilated COVID-19 patients in general. This is further supported by the fact that the majority of EIT measurements during SMV are performed within seven days after starting SMV, meaning the majority of the included patients could not have been considered easy- or difficult-to-wean [34], minimizing the chance that inclusions in the present study were included based on their ability to wean successfully. Nevertheless, our findings remain to be studied in a non-COVID-19 population and in a difficult to wean population on SMV.

EIT itself only covers 5–10 centimetres of the axial section of the chest, limiting the generalization of the EIT outcomes for the more basal or apical lung tissue. Also, the spatial resolution of EIT, which is 32 by 32 pixels, is low compared to other imaging modalities (e.g., computed tomography or magnetic resonance imaging). Moreover, tidal volumes of patients subjected to SMV may show high variability within a specific PEEP level, leading to a wide range of parameter outcomes per PEEP level. In the present study, the parameter outcomes are averaged for each PEEP level in order to correct for this variability. It should be kept in mind that outcomes from highly variable breaths within one PEEP level may underestimate or overestimate the true clinical and mechanical outcomes. Last, in our analysis, we calculated pendelluft based on the absolute impedance shift of a single pixel relative to the peak global impedance, which is a relative outcome. After that, the relative amount of pendelluft can be converted to an absolute volume by multiplying the above-mentioned relative outcome by the total tidal volume adapted from the ventilator. Since the EIT belt does not cover the complete lungs and we use the measured tidal volumes from the ventilator, the quantification of pendelluft in the present study may be overestimated.

We only performed PEEP steps of approximately 30 to 60 s and cannot rule out any long-term changes in tidal volume, end-tidal CO_2_, CAR, pendelluft or recruitment rate [35].

### Strengths and clinical implications

Human studies regarding the effects of PEEP and the determination of a universally accepted PEEP titration approach in SMV remain scarce. Therefore, we studied the added value of EIT in this specific matter. The present study shows that PEEP titration in spontaneous mechanical ventilation by EIT is feasible and is linked to improved pulmonary mechanics in COVID-19-related ARDS lungs. It does not require additional bedside procedures compared to EIT-guided PEEP titration in controlled mechanical ventilation. On average, higher PEEP may be required considering pulmonary mechanics in COVID-19-related ARDS patients in an early stage of spontaneous mechanical ventilation. Furthermore, it should be noted that after EIT-based PEEP titration, each patient’s ventilation strategy may need to be personalized according to the patient’s clinical status. Due to the observational nature of the present study, the effects of EIT-guided PEEP in SMV on longer-term clinical outcomes (e.g., PaO_2_/FiO_2_, ventilation days, and extubation success) could not be assessed in the present study and should be evaluated in future research.

## Conclusion

This study presents the first application of our previously developed EIT algorithm for performing a novel method of PEEP titration in acute respiratory distress syndrome patients on spontaneous mechanical ventilation. The EIT-based optimal PEEP threshold of approximately 5% cumulative collapse is related to improved pulmonary mechanics (i.e., less pendelluft, cyclic alveolar recruitment, and alveolar collapse and improved alveolar recruitment). Remarkably, at this threshold, higher PEEP was required compared to baseline. These findings encourage prospective interventional studies, focusing on the effects of EIT-guided PEEP titration by the regional peak flow algorithm on long-term mechanical and clinical outcomes in spontaneous mechanical ventilation.

## Electronic supplementary material

Below is the link to the electronic supplementary material.

Supplementary Material 1

## Data Availability

The data that support the findings of this study are available from the corresponding author upon reasonable request.

## Abbreviations

APACHE: Acute physiology and chronic health evaluation
ARDS: Acute respiratory distress syndrome
CAR: Cyclic alveolar recruitment
CL: Cumulative alveolar collapse rate
EIT: Electrical impedance tomography
etCO_2_: End-tidal carbon dioxide
ICU: Intensive Care Unit
OD: Cumulative alveolar overdistension rate
P0.1: Airway occlusion pressure at 100 milliseconds
PBW: Predicted body weight
PEEP: Positive end-expiratory pressure
RPF: Regional Peak Flow
SMV: Spontaneous mechanical ventilation
SOFA: Sequential Organ Failure Assessment
TV: Tidal volume per kilogram predicted body weight
