# Supplementary material for: A novel positive end-expiratory pressure titration using electrical impedance tomography in spontaneously breathing acute respiratory distress syndrome patients on mechanical ventilation: an observational study from the MaastrICCht cohort
Source: J Clin Monit Comput. 2024 Aug 28;39(1):127–39. doi: 10.1007/s10877-024-01212-8 (PMC11821668; doi:10.1007/s10877-024-01212-8)
Supplement: Supplementary file 1 — Supplementary Material 1 [file 10877_2024_1212_MOESM1_ESM.docx]

**Supplemental Information**

**Journal of Clinical Monitoring and Computing**

**Standardized positive end-expiratory pressure titration using electrical impedance tomography in spontaneously breathing acute respiratory distress syndrome patients on mechanical ventilation: an observational study from the MaastrICCht cohort**

S.J.H. Heines^*^, S.A.M. de Jongh*, F.H.C. de Jongh, R.P.J. Segers, K.M.H. Gilissen, I.C.C. van der Horst, B.C.T. van Bussel, D.C.J.J. Bergmans

*****Both authors contributed equally

Correspondence to: Serge Heines

Department of Intensive Care Medicine

Maastricht University Medical Center+

P. Debyelaan 25

P.O. Box 5800, 6202 AZ Maastricht, The Netherlands

Email: S.Heines@mumc.nl

ORCID iD: 0000-0001-7672-4277

**Table S1**. Patient characteristics for the group with EIT in SMV and the rest of the invasive mechanically ventilated MaastrICCht cohort

| Patient characteristics^¶^ | SMV (n=25) | PCV (n=207) | *p*-value |
| --- | --- | --- | --- |
| Age, year | 66.0 [59.5 – 74.0] | 65.0 [57 - 73] | 0.497**^†^** |
| Gender, men | 20 (80.0%) | 158 (74.9%) | 0.574^‡^ |
| Body mass index, kg/m^2^ | 28.7 [26.4 – 30.6] | 27.6 [24.9 – 30.8] | 0.288**^†^** |
| Chronic lung disease, N | 4 (16.0%) | 33 (15.6%) | 0.963^‡^ |
| APACHE II score, points | 14.0 [12.5 – 17.3] | 15.0 [13.0 – 18.0] | 0.478**^†^** |
| SOFA score, points | 10.0 [9.5 – 12.3] | 10.0 [7.0 – 12.0] | 0.279**^†^** |
| Intubation, N | 22 (88.0%) | 178 (84.4%) | 0.632^‡^ |
| FiO_2_, % | 80 [50 – 100] | 70 [60 - 100] | 0.803**^†^** |
| PaO_2_/FiO_2_, mmHg | 13.0 [10.0 – 20.0] | 15.0 [11.0 – 21.0] | 0.354**^†^** |
| PaO_2_, mmHg | 9.5 [8.8 – 10.0] | 9.6 [8.2 – 11.0] | 0.590**^†^** |
| PaCO_2_, mmHg | 4.9 [4.5 – 5.8] | 5.4 [4.4 – 6.3] | 0.343**^†^** |
| pH | 7.48 [7.35 – 7.51] | 7.43 [7.28 – 7.49] | 0.049^*^**^†^** |
| Respiratory Rate, min^-1^ | 26 [23 – 29] | 26 [23 – 32] | 0.212**^†^** |
| Mean Arterial Pressure, mmHg | 107 [87 – 122] | 99.0 [91 – 111] | 0.622**^†^** |
